# The lack of the Celf2a splicing factor converts a Duchenne genotype into a Becker phenotype

**DOI:** 10.1038/ncomms10488

**Published:** 2016-01-22

**Authors:** J. Martone, F. Briganti, I. Legnini, M. Morlando, E. Picillo, O. Sthandier, L. Politano, I. Bozzoni

**Affiliations:** 1 Department of Biology and Biotechnology Charles Darwin, Sapienza University of Rome, P.le A. Moro 5, Rome 00185, Italy; 2 Department of Experimental Medicine, Cardiomyology and Medical Genetics, Second University of Napoli, I Policlinico, Piazza Miraglia, Napoli 80138, Italy; 3 Center for Life Nano Science@Sapienza, Istituto Italiano di Tecnologia, Viale Regina Elena 291, Rome 00161, Italy; 4 Institute Pasteur Fondazione Cenci-Bolognetti, Sapienza University of Rome, P.le A. Moro 5, Rome 00185, Italy

## Abstract

Substitutions, deletions and duplications in the *dystrophin* gene lead to either the severe Duchenne muscular dystrophy (DMD) or mild Becker muscular dystrophy depending on whether out-of-frame or in-frame transcripts are produced. We identified a DMD case (GSΔ44) where the correlation between genotype and phenotype is not respected, even if carrying a typical Duchenne mutation (exon 44 deletion) a Becker-like phenotype was observed. Here we report that in this patient, partial restoration of an in-frame transcript occurs by natural skipping of exon 45 and that this is due to the lack of Celf2a, a splicing factor that interacts with exon 45 in the *dystrophin* pre-mRNA. Several experiments are presented that demonstrate the central role of Celf2a in controlling exon 45 splicing; our data point to this factor as a potential target for the improvement of those DMD therapeutic treatments, which requires exon 45 skipping.

## Abstract (web summary)

Muscular Dystrophy can be caused by mutations in the *dystrophin* gene, causing the severe Duchenne form or the mild Becker form depending on if the transcript is in or out-of-frame. Here the authors identify a Duchenne-type mutation that gives a Becker-like phenotype due to skipping of exon 45.

## Graphical abstract

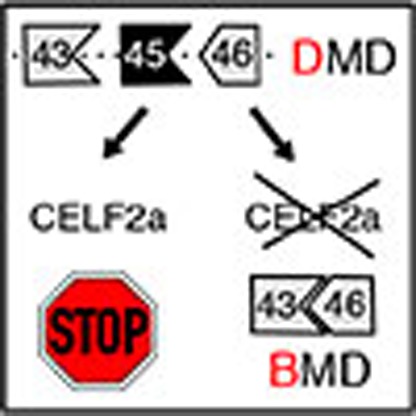

Duchenne muscular dystrophy (DMD) is a rare neuromuscular disease characterized by substitutions, deletions and duplications in the *dystrophin* gene leading to frameshift and premature translation termination^1^. Dystrophin is a cytoskeleton protein which stabilizes the dystrophin-associated protein complex, required for muscle fibre integrity^2^, and controls gene expression through nNOS activation and nitric oxide-mediated HDAC2 inactivation^3^,^4^,^5^. At variance with DMD, the milder form of the disease, the Becker muscular dystrophy (BMD), is caused by mutations, including long deletions, which do not perturb the open reading frame (ORF) integrity and produce shorter and partially functional dystrophin isoforms. Deletions in the *dystrophin* gene are found in ∼68% of DMD patients, with the region including exons 44–55 being the most commonly mutated, together with the one encompassing exons 2–20 (ref. 6). However, the severity of the pathology is not always predictable by exon deletion, suggesting that each patient has specific deletion breakpoints that may differentially affect splicing. Moreover the ‘reading frame model', in which BMD patients produce a semi-functional, internally deleted dystrophin protein, whereas DMD patients have no detectable protein, is not always respected^7^,^8^. Uncertainty in predicting the Duchenne/Becker pathology indicates that the clinical picture should always be accompanied by a detailed molecular analysis, including transcript structure and dystrophin-associated proteins. Several data indicated that altered splicing patterns in a collection of BMD patients do not directly result from the gene defect but probably derive from modifications in *trans*- rather than *cis*-acting factors^9^.

The fact that BMD patients carrying extended deletions display only mild phenotypes, suggested that manipulating the splicing of the dystrophin pre-mRNA, to induce the skipping of specific exons and restore a correct reading frame, could be a powerful strategy for DMD treatment^10^,^11^,^12^,^13^. This approach has recently entered into clinical trials through the use of antisense oligonucleotides preventing exon recognition by the splicing machinery^14^,^15^.

In this work we have studied a DMD case (GSΔ44), in which the correlation between genotype and phenotype is not respected: even if the genetic analysis of the *dystrophin* gene locus showed a deletion of exon 44, which should normally lead to DMD, the patient is now 18 years old and can still walk independently. Molecular analyses on both GSΔ44 biopsy and primary myoblasts showed the presence of tiny but consistent dystrophin levels due to endogenous skipping of exon 45. We describe here that this phenotype is due to the lack of the Celf2a protein isoform, that we found to enhance exon 45 inclusion.

## Results

### A DMD patient produces dystrophin by natural exon skipping

An individual (GSΔ44) with a DMD genotype (deletion of exon 44 which leads to an out-of-frame transcript) was under study since, quite surprisingly, his clinical and behavioural tests were in agreement more with a Becker phenotype, even if of a severe type. Supplementary Table 1 summarizes the timed tests from the age of 5 to 18: while DMD patients normally lose ambulance around the age of 12 and need ventilatory support at their late teens, GSΔ44 is 18 years old and, even if he has lost the ability to stand up from the ground, he can walk and breath normally.

**Figure 1:**
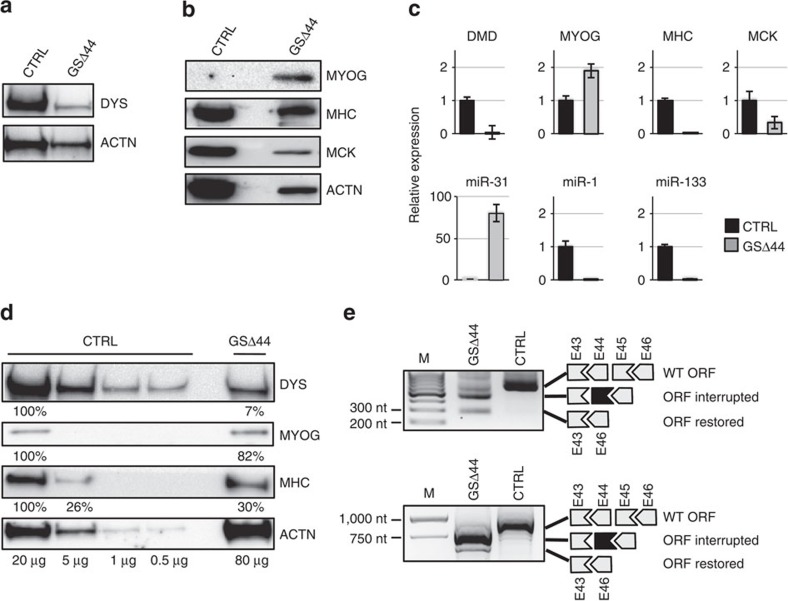
Expression of dystrophin and myogenic markers in GSΔ44 biopsy and primary myoblasts. (**a**,**b**) Western blot on proteins (20 μg) extracted from human muscle biopsies of a healthy control (CTRL) and from GSΔ44 probed with antibodies against dystrophin (DYS, **a**), or myogenin (MYOG), MHC and muscle creatine-kinase (MCK, **b**). Actinin (ACTN) was used as a loading control. (**c**) Quantitative RT–PCR of myogenic markers and myomiRs performed on RNA extracted from healthy control (CTRL) and GSΔ44 muscle biopsies. Bar plots show relative expression levels with respect to control set to 1: values were normalized with GAPDH and hypoxanthine phosphoribosyltransferase (HPRT) for mRNAs and with U6 and miR-16 for miRNAs. Bars indicate a confidence level of 95%. (**d**) Western blot on proteins extracted from healthy control (CTRL) and GSΔ44 primary myoblasts upon 10 days of shift to differentiation conditions and probed with antibodies against dystrophin (DYS), myogenin (MYOG) and MHC. The amount of proteins loaded is indicated below each lane. Actinin (ACTN) was used as a loading control. (**e**) Nested RT–PCR performed on RNA from biopsy samples (upper panel) and differentiated myocytes obtained as in **d** (lower panel); 20 ng of cDNA were amplified with primers located in exons 42 and 53 and then with primers in exons 43 and 46 (upper panel) and in exons 43 and 48 (lower panel). A schematic representation of the WT, interrupted and restored ORFs is shown on the side. M, molecular weight marker: GeneRuler 100 bp (Thermo Scientific) for the upper panel and GeneRuler 1 kb (Thermo Scientific) for the lower panel; sizes are shown on the side.

A muscle biopsy, performed at the age of 15, indicated the unexpected presence of dystrophin at low but well detectable levels (Fig. 1a). As expected for a dystrophic sample^16^ regeneration was occurring: the early myogenic factor myogenin (MYOG) was still expressed, while the terminal differentiation markers, myosin heavy chain (MHC) and muscle creatin kinase (MCK)^17^, were lower with respect to the biopsy from a healthy control (Fig. 1b,c). Regeneration was also indicated by sustained expression of the proliferative miR-31 and by reduced levels of miR-1 and miR-133, markers of mature muscle fibres^18^ (Fig. 1c). Since muscle biopsies can vary considerably on the basis of the inflammatory and fibrotic state of the tissue, we derived a culture of primary myoblasts and analysed dystrophin expression upon differentiation in low serum, in parallel to control myocytes derived from a healthy individual. In agreement with the biopsy sample*, in vitro* differentiation confirmed the ability of GSΔ44 myocytes to produce dystrophin (Fig. 1d). A scaled dilution of proteins from controls indicated that these levels corresponded to ∼7% of those found in healthy myocytes. Interestingly, such low levels of dystrophin paralleled a slower timing of differentiation^16^,^19^: in fact, myogenin expression was still detectable at day 10, while MHC was reduced in comparison with control (Fig. 1d). This is in line with the notion that dystrophic cultures show a slower progression into the differentiation process^16^, with BMD having on average a less severe differentiation phenotype than DMD^20^,^21^.

A detailed mapping of the GSΔ44 deletion revealed a 4065, bp deletion, encompassing exon 44, with the fusion between nucleotide 69586 of intron 43 and nucleotide 3038 of intron 44 (Supplementary Fig. 1a–c and Supplementary Methods). Therefore, we confirmed the DMD genotype of GSΔ44.

Reverse transcription PCR (RT–PCR) analysis on RNA from the GSΔ44 biopsy and from myocytes derived from primary myoblasts indicated that in addition to the lack of exon 44, a small fraction of transcripts also skipped exon 45 (Fig. 1e). Sequencing of this product confirmed the skipping of exon 45 and the proper fusion between exon 43 and exon 46 (not shown), giving rise to an in-frame transcript coding for a shorter but functional protein^21^,^22^. Notably, the skipping of exon 45 is currently conceived as a possible approach for the therapeutic treatment of Δ44 deletions and for those DMD mutations that through exon 45 skipping could recover the correct reading frame^22^. The presence of sub-optimal splice junctions in exon 45 of the *DMD* gene of GSΔ44 was excluded by PCR amplification and sequence analysis of the genomic DNA (data not shown).

**Figure 2:**
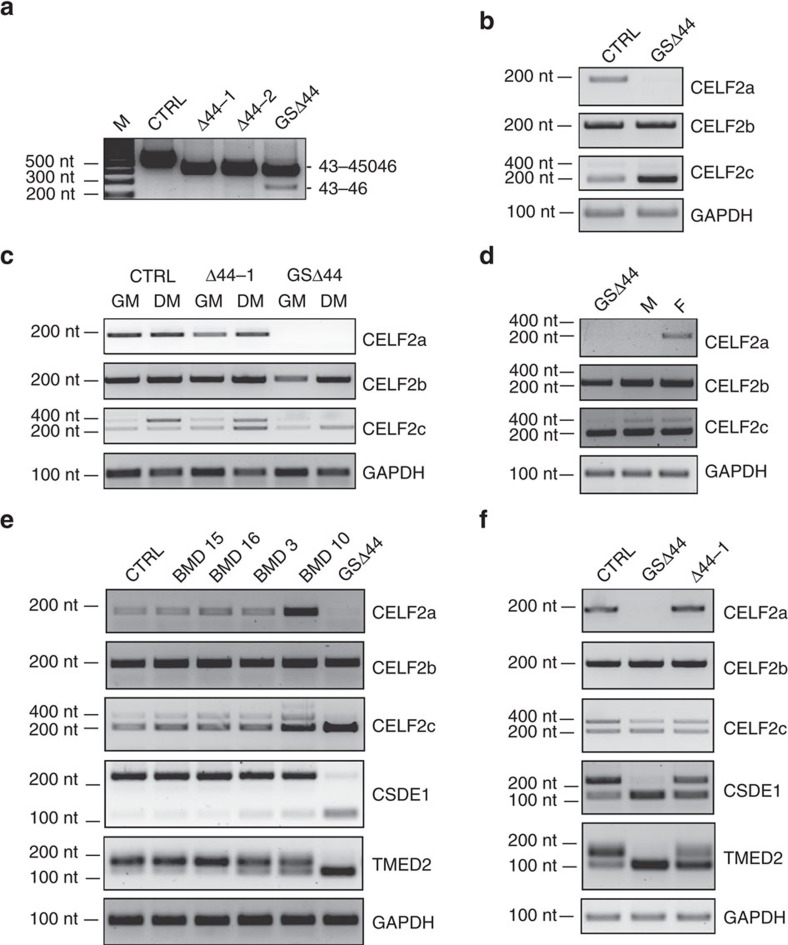
GSΔ44 lacks the CELF2a isoform. (**a**) Fibroblasts from control (CTRL), two Δ44 unrelated DMD (Δ44-1 and Δ44-2) and GSΔ44 biopsies were *trans-*differentiated into myocytes via infection with a lentivirus containing a MyoD-expression cassette. After 10 days in differentiation medium, RNA was extracted and RT–PCR was performed with primers located in exons 43 and 46. M, molecular weight marker: GeneRuler 100 bp (Thermo Scientific). (**b**) RT–PCR for the three CELF2 isoforms on RNA from control (CTRL) and GSΔ44 biopsies. (**c**) MyoD-transduced fibroblasts from control (CTRL), Δ44-1 and GSΔ44 were maintained in growth conditions (GM) or shifted to differentiation medium (DM) for 10 days. RNA was extracted and analysed by RT–PCR with oligos specific for the three CELF2 isoforms. (**d**) MyoD-transduced fibroblasts from the mother (M) and father (F) of GSΔ44 were induced to differentiate and RNA analysed by RT–PCR for the three CELF2 isoforms. (**e**) RT–PCR for the three CELF2 isoforms and for CSDE1 and TMED2 mRNAs on RNA from control (CTRL), GSΔ44 and 4 different BMD biopsies (BMD-3, BMD-10, BMD-15 and BMD-16). (**f**) RT–PCR for the three CELF2 isoforms and for CSDE1 and TMED mRNAs on RNA from control (CTRL), Δ44-1 and GSΔ44 *trans*-differentiated myocytes. In all experiments GAPDH was used as control. Molecular weight marker: GeneRuler 100 bp (Thermo Scientific).

Because of the limited ability of *in vitro* propagation of primary myoblasts, fibroblasts derived from the GSΔ44 biopsy and *trans-*differentiated with MyoD towards the myogenic lineage^21^ were also used. These cells produced exon 45 skipping and dystrophin synthesis similarly to differentiated primary myoblasts, even if with a delayed time of expression (Fig. 2a and Supplementary Fig. 2a). Notably, two different *trans-*differentiated Δ44 DMD myocyte cultures, carrying unrelated deletions around exon 44 (Δ44-1 and Δ44-2), did not show any detectable exon 45 skipping (Fig. 2a).

### GSΔ44 myoblasts lack the Celf2a splicing factor

RNA-seq experiments, performed on healthy control, GSΔ44 and Δ44-1 myoblasts and myocytes (deposited at Gene Expression Omnibus, GSE70389), were queried for the abundance of splicing enhancers (SRSF1, SRSF2, SRSF5 and SRSF6) whose potential binding motifs were identified on exon 45 by using the ESE Finder tool (ref. 23 and Supplementary Fig. 2b). This analysis indicated that abundance of such mRNAs did not vary among the different samples; finding that was validated by RT–PCR (Supplementary Fig. 2c). Therefore, none of these factors could be considered a good candidate for the expected phenotype.

Other possible candidates, among the factors previously known to change during muscle differentiation, were searched. Among them, the CUGBP, Elav-like family member 2 (Celf2) protein was demonstrated to regulate various processes including alternative splicing, editing, RNA stability and translation^24^,^25^,^26^,^27^,^28^,^29^ and suggested to participate in dystrophin splicing^9^. CELF2 is composed of three isoforms differing by their first exon (exons 1a, 1b and 1c)^9^, that are here termed CELF2a, b and c, respectively (Supplementary Fig. 2d); moreover, CELF2a has a variant isoform consisting in the alternative use of a 18 nt extended exon 11 (ref. 28). Analysis of CELF2 transcripts in the RNA-seq of control, GSΔ44 and Δ44-1 samples indicated that while the CELF2b and CELF2c isoforms were expressed in all samples, CELF2a was completely absent in GSΔ44 both in growth (GM) and differentiation (DM) conditions.

The absence of CELF2a mRNA, was validated by RT–PCR both in the biopsy sample (Fig. 2b) and in myoblasts (GM) and myocytes (DM) of GSΔ44 (Fig. 2c). Notably, all three isoforms were instead present in control and Δ44-1 cells (Fig. 2c). Western blot analysis with commercially available antibodies indicated their inability to distinguish between the three different isoforms (Supplementary Fig. 2e); therefore, in following analyses CELF2 isoform expression was always tested at the RNA level.

When analysing *trans-*differentiated fibroblasts of GSΔ44's parents, it was interesting to find that while CELF2a is present in the father, the mother specifically lacks this isoform (Fig. 2d) and displays skipping of exon 45 (Supplementary Fig. 2f). In her case, the skipped product is present at very low levels since exclusion of exon 45 would produce an out-of-frame transcript that undergoes degradation through the NMD pathway.

A 1.3 kb DNA sequence upstream the TSS and 200 nt of the first exon/intron of *CELF2a* was sequenced in the GSΔ44 DNA. We identified only two variants of annotated single-nucleotide polymorphisms (chr10:11046386 T/C, chr10:11047206 C/G of the hg19 human assembly), one of which was laying at the border of a CpG island; neither sequence corresponded to obvious transcriptional factor binding sites as checked on the Encode Transcriptional factors binding tracks at UCSC genome browser and on TransFac database. To check whether methylation could affect CELF2a expression, GSΔ44 myoblasts were treated for 7 days with 5-azacytidine in parallel with control myoblasts. No rescue of CELF2a expression was obtained in treated versus untreated GSΔ44 cells, suggesting that likely promoter methylation is not the cause of CELF2a lack in these cells (Supplementary Fig. 2g). Also in control cells, 5-azacytidine treatment did not affect CELF2a levels (Supplementary Fig. 2g and Supplementary Methods).

To detect similar exon skipping events possibly related to the absence of CELF2a, the RNAseq data obtained from control, Δ44 and GSΔ44 myoblasts and myocytes were analysed by MATS (ref. 30). Ratios of exon inclusion/skipping were compared in GSΔ44 versus Δ44-1 and in GSΔ44 versus control (Supplementary Table 2 and Supplementary Data 1). To exclude exon skipping events related to the pathological state or to altered differentiation of DMD cells, we selected only those that were concordant and significant in both GSΔ44 versus control and GSΔ44 versus Δ44-1. Out of 29 genes, correlating with the lack of CELF2a (Supplementary Table 2), five transcripts exhibited alternative splicing events in differentiated myocytes and two of them were experimentally validated, CSDE1 and TMED2. In comparison with control and Δ44-1, GSΔ44 shows the preferential accumulation of skipped isoforms for both CSDE1 and TMED2 transcripts (Supplementary Fig. 2h,i).

Four unrelated BMD biopsies^21^ (Supplementary Fig. 2j) were then analysed; they all expressed CELF2a and displayed the same CSDE1 and TMED2 isoforms as the control sample (Fig. 2e). The same isoforms' pattern was also obtained in Δ44-1 myocytes (Fig. 2f). In contrast, the GSΔ44 biopsy and myocytes, besides lacking CELF2a, showed only the skipped isoforms for both CSDE1 and TMED2 (Fig. 2e,f). Notably, while the isoforms present in control and BMD are found in mature muscle fibres (Supplementary Table 2), those present in GSΔ44 correspond to more ubiquitous species^31^. In fact, comparison of CSDE1 and TMED2 in wild-type (WT) and GSΔ44 fibroblasts and myoblasts, indicated that only the unskipped species are the myocyte-specific isoforms (Supplementary Fig. 2k). The skipped splicing variants detected in GSΔ44 do not have premature stop codons and maintain well-defined protein coding ability even if they are not specific for muscle cells. From these data it cannot be excluded whether any of these changes in isoform ratio could have secondary detrimental effects, even though GSΔ44 does not show any adverse effect in any other specific physiological activity.

### Celf2a affects exon 45 splicing

**Figure 3:**
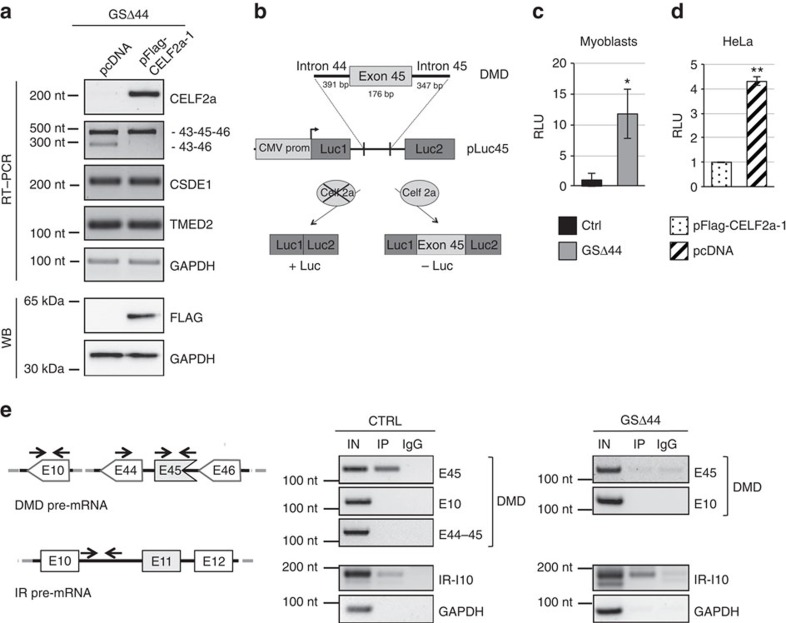
CELF2a favours exon 45 inclusion. (**a**) GSΔ44 *trans-*differentiated myocytes were transfected with an empty vector (pcDNA) or with a Flag-CELF2a construct (pFlag-CELF2a-1). Cells were shifted to DM medium for 10 days and the RNA analysed by RT–PCR for Flag-CELF2a expression, for exon 45 skipping (43–46) and for CSDE1 and TMED2 isoforms (upper panel). The expression of the exogenous Flag-CELF2a was tested by western blot with anti-FLAG antibodies (lower panel). GAPDH was used as loading control. (**b**) Schematic representation of the pLuc45 construct: the *DMD* gene region encompassing exon 45 plus portions of the flanking introns was cloned into the intron of pcDNA3.1-Luc. This plasmid contains the CMV promoter driving the expression of a Firefly luciferase pre-mRNA that produces luciferase only upon splicing (lower part). (**c**) The Bar plots show the relative luminescence units (RLU) of Firefly luciferase in control (black bar) and GSΔ44 (grey bar) myoblasts transfected with pLuc45 and incubated 48 h. The values were normalized for Renilla luciferase activity derived from a co-transfected pRL-TK plasmid. Error bars represent standard error from two independent experiments. *P<0.05. (**d**) The Bar plots show the RLU of Firefly luciferase in HeLa cells transfected with pLuc45 and either pFlag-CELF2a-1 (dotted bar) or pcDNA (dashed bar) and incubated 48 h. The values were normalized for Renilla luciferase activity derived from a co-transfected pRL-TK plasmid. Error bars represent s.e.m. from three independent experiments. **P<0.01. (**e**) Left panel: schematic representation of part of the dystrophin (DMD) and insulin receptor (IR) pre-mRNAs. Arrows indicate the positions of the oligos used. Right panels: control (CTRL) and GSΔ44 myocytes were UV crosslinked and subjected to nuclear-cytoplasmic fractionation. Nuclear extracts were immunoprecipitated by anti-CELF2 (lane IP) and control IgG antibodies. The presence of the regions indicated on the right (E45, E44–45 and E10 for DMD; IR-I10 for IR) was analysed by RT–PCR. GAPDH was used as negative control. ‘IN' samples represent 10% of the total nuclear extract. Molecular weight marker: GeneRuler 100 bp (Thermo Scientific). CMV, cytomegalovirus.

We next tested whether rescue of Celf2a in GSΔ44 could affect exon 45 splicing. A Flag-CELF2a construct (pFlag-CELF2a-1) or an empty vector (pcDNA) were transfected into GSΔ44 myoblasts and exon 45 skipping was analysed upon induction of differentiation. Efficient expression of CELF2a was confirmed in GSΔ44 myocytes both at the RNA and protein levels (Fig. 3a). Notably, under these conditions, exon 45 skipping was prevented (Fig. 3a). The same result was obtained when using a plasmid encoding for the CELF2a variant, lacking the 18 nt extension in exon 11 (pCELF2a-2, Supplementary Fig. 3a). Interestingly, the overexpression of either forms of CELF2a did not produce any change in the splicing pattern of CSDE1 and TMED2 (Fig. 3a) indicating that Celf2a has no effect on these two substrates. These results open the interesting question whether CELF2a, together with CSDE1 and TMED2, are on a common pathway perhaps responding to a common upstream effector.

To further analyse Celf2a splicing activity, Δ44-1 myoblasts were infected with a lentiviral construct expressing shRNAs against CELF2a and induced to differentiate for 10 days in the presence of anti-CELF2a LNA GapmeRs. With respect to a 30% decrease of CELF2a, a little amount of exon 45 skipping was observed, indicating again the ability of this factor to impinge specifically on exon 45 splicing in a myogenic environment (Supplementary Fig. 3b and Supplementary Methods).

Celf2a activity was also tested on an artificial construct (pLuc45, Fig. 3b) containing exon 45 and portions of the flanking regions cloned inside the intron of pcDNA3.1-Luc (refs 32, 33). From pLuc45, luciferase can be produced only when exon 45 is skipped. When GSΔ44 myoblasts were transfected with pLuc45, a 10-fold increase in luciferase activity with respect to control cells was obtained (Fig. 3c). These data indicate that the absence of CELF2a leads to exon 45 skipping also in this artificial construct and that the regions included are sufficient to respond to Celf2a activity. The effects of CELF2a absence were also tested in a cellular context devoid of CELF2a, such as HeLa cells (Supplementary Fig. 3c). Therefore, pLuc45 was transfected into HeLa cells together with pFlag-CELF2a or with an empty vector as control (pcDNA). The results show that while very little luciferase activity is produced upon ectopic expression of CELF2a (Supplementary Fig. 3d), in control cells luciferase levels increase by four-fold (Fig. 3d).

Finally, to test whether Celf2a interacts with the region containing exon 45, a cross-linking immunoprecipitation (CLIP) assay was performed on nuclear RNA from control and GSΔ44 myocytes. As a positive control, the region inside intron 10 of the insulin receptor gene (*IR*), previously reported to bind Celf2c (refs 25, 34), was used. Figure 3e shows that, in control myocytes (panel CTRL), Celf2 antibodies are able to immunoprecipitate the region containing exon 45 of the dystrophin pre-mRNA (E45) as well as a specific region inside intron 10 of the IR transcript (IR-I10). No interaction was instead detected on regions containing exon 10 (E10) of the dystrophin pre-mRNA or when amplification of the spliced exons 44–45 (E44–45) region was performed. Notably, when CLIP was performed on GSΔ44 myocytes (panel GSΔ44), no signal was found on the dystrophin exon 45 containing region, while interaction was detected on intron 10 of the IR transcript (Fig. 3e). Since GSΔ44 myocytes express only the CELF2b and c isoforms, these results indicate that exon 45 interaction in control cells is specifically due to CELF2a. In conclusion, these data indicate that the Celf2a isoform is able to interact with the dystrophin pre-mRNA at the level of exon 45 and to enhance its inclusion during the splicing reaction.

## Discussion

The exon skipping strategy, as a therapeutic approach for DMD, relies on the concept of converting severe Duchenne phenotypes into milder Becker ones. In line with this, several studies have shown that the disease course of BMD patients with a mutation mimicking a 'skipped' DMD mutation is relatively mild even though BMD individuals display a large range of phenotypes^35^, likely reflecting the ability of shorter dystrophin proteins to accomplish only a subset of the different functions of the WT full-length form^8^ and to be able to produce different amounts of dystrophin. This strong variability stands for individual heterogeneity likely including the specific deletion as well as possible trans-acting factors^9^.

Moreover, one of the major concerns about the exon skipping approach resides on how much dystrophin should be required in order to be of therapeutic benefit. Here we show a DMD patient case of a 18-year-old boy, with a Δ44 mutation; however, his clinical features, are in net contrast with the common lost of deambulation ability of DMD patients occurring around the first decade of age. Surprisingly, GSΔ44 myocytes, through spontaneous exon skipping, produce 7% of dystrophin with respect to control cells, indicating that this amount of dystrophin is enough to alleviate the severity of the disease. Therefore, GSΔ44 is an exemplary human case indicating that even very low levels of dystrophin might provide milder dystrophic conditions. GSΔ44 presents an intermediate phenotype between a typical DMD patient and a Becker individual, who can be asymptomatic for several decades; in fact, GSΔ44 walks independently even if has lost the ability to stand up from the ground. This is in line with the finding that levels of dystrophin slightly higher than those of GSΔ44 were found in a large cohort of BMD individuals^35^ and required in mice to slow down or prevent disease progression and improve overall muscle function^36^,^37^.

By transcriptome analysis of GSΔ44 myocytes, we discovered the absence of CELF2a, a specific isoform of the Celf2 splicing factor and we were able to correlate the absence of this product with exon 45 skipping occurring in GSΔ44. CELF2 mRNA is expressed at variable levels in BMD/DMD skeletal muscles and its role in splicing regulation was previously suggested^9^. Celf2 belongs to the CELF/Bruno-like family of RNA-binding proteins that, in mammals, contains six members. This family of highly conserved proteins is involved in regulation of cell-specific and developmentally regulated alternative splicing^29^. The highest expression of CELF2 is found in heart, skeletal muscle and brain and single-nucleotide mutations in this gene have been correlated with a familial form of arrhythmogenic right ventricular^28^.

Since no mutations were found around the promoter and first exon/intron regions of the GSΔ44 *CELF2a* gene, it is likely that the genetic cause for its absence resides on upstream factors regulating its biogenesis or to a still unidentified epigenetic control. As other transcripts appear to be specifically affected in GSΔ44 myocytes, it will be interesting to analyse whether they are part of a common regulatory pathway specifically impinging on muscle homeostasis.

Our data suggest that CELF2a inhibition could represents an interesting new strategy to be considered for improving DMD treatment, at least in those cases where the skipping of exon 45 would result in the recovery of mRNA with an ORF. A relevant challenge for the future would be to design and set up genetic or pharmacological treatments for interfering with Celf2a activity; moreover, these results point to the relevance of studying the genomic milieu of different patients in order to facilitate the clinical development of personalized therapies.

## Methods

### Case report

The proband is a 18-year-old boy, the first child of non-consanguineous Italian healthy parents. There was no family history of autoimmune or musculoskeletal disorders in the family. The birth was at term, by caesarean delivery. The baby presented neonatal jaundice treated by phototherapy. The child has never crawled and has acquired independent walking at 18 months. At 1 month of age he presented intolerance to breast milk so he began to take formula. At 7 months he presented an episode of bronchospasm that required hospitalization at the department of pediatrics of the local hospital. During hospitalization, laboratory tests pointed out high levels of transaminases (GOT 797 U l^−1^ and GPT 266 U l^−1^) and creatin kinase (12622 U l^−1^). Because this elevation, the family has been addressed at the Cardiomyology and Medical Genetics of Second University of Naples suspecting a muscle disorder, where he arrived at the age of 8 months. At the first control, the baby did not show pseudo-hypertrophy of the calves, but given the high values of the creatin kinase, a blood sample was carried out, with the mother's consent, to perform the molecular analysis of the *dystrophin* gene. The analysis showed the isolated deletion of the exon 44, an out-of-frame mutation consistent with a diagnosis of DMD. Since then, the child underwent regular checks every 6 months, by the same clinical team. Despite the prognosis, the evolution of the disease was very mild, at the age of 5 years the boy was still able to stand up from the floor in 5 s, though showing the Gowers manoeuvre, and to climbing stairs without support in 2 s. The electrocardiogram showed signs of postero-lateral fibrosis, a picture typical of dystrophinopathic cardiomyopathy^38^ so that the administration of steroids (Deflazacort −0,75 mg kg^−1^ day^−1^) and Ace-inhibitors (Fosinopril 5 mg day^−1^) was started, according to standard protocol.

At the last control—at the age of 18 years—the boy walks independently, with a waddling gait, but shows some difficulty in getting up from a chair and climbing stairs. North Star (NSAA) has a value of 8/34 and he was able of walking 252 m in 6 min (6MWT). The electrocardiogram showed a progression of the fibrosis, while echocardiography showed normal diameters and volumes of cardiac chambers, normal thickness of septum and left posterior wall and a normal ejection fraction. There was a mild reduction of the vital capacity values (FVC 2610, ml, 61%).

### Skeletal muscle biopsies

BMD-3, BMD-10, BMD-15, BMD-16 and control skeletal muscle biopsies were obtained, with informed consent, from the Laboratory of Molecular Medicine, Department of Neuroscience, Bambino Gesù Children's Hospital, Rome whereas SGΔ44 biopsy was obtained from the EuroBiobank and Telethon Neuromuscular Biobank of Second University of Napoli.

### Isolation of myoblasts from muscle biopsies

Muscle tissue was minced and placed at 37 °C for 45 min, with gently shaking, in 20 ml of phosphate-buffered saline (PBS)-collagenase solution (1 mg ml^−1^). Resuspended solution was then passed throught a 70 μm filter. After centrifugation (1500, r.p.m. for 10 min at 4 °C) the pellet was resuspended in RPMI, 15% FBS, 1X P/S, plated in collagenate plates and incubated at 37 °C with 5% of CO_2_.

### Cell cultures and treatments

WT (9208), Δ44-1 (9981) (from the Telethon Network of Genetic Biobanks) and GSΔ44 human primary myoblasts were cultured in Human skeletal muscle growth medium (PromoCell) and grown in a humidified incubator, at 5% CO_2_ and 37 °C. Cells were induced to differentiate with human skeletal muscle differentiation medium (PromoCell). Transient transfection of plasmids was carried out with the use of Lipofectamine-2000 (Invitrogen) according to the manufacturer's specifications.

### Reprogramming of fibroblasts into myoblasts

Conversion of fibroblasts into myoblasts was achieved by ectopic expression of *MyoD* gene^39^,^40^ through lentiviral infection.

### Virus preparation and cell transduction

Viruses were prepared as described^33^. Cells were transduced with 5 × 10^6^ lentiviral particles for 5 h and harvested after 10 days of differentiation.

### DNA and RNA preparation

Cells were harvested in QIAzol Lysis Reagent (Qiagen) and biopsies were homogenized with a rotor-stator homogenizer in the presence of QIAzol Lysis Reagent (Qiagen). RNA was extracted by miRNeasy (Qiagen), following manufacturer's specifications; Genomic DNA was extracted from the organic phase (interphase) of QIAzol Lysis Reagent (Qiagen) after RNA preparation. Concentrations were assessed with Nanodrop ND-1000 Spectrophotometer (CELBIO; Pero, Milan, Italy).

### Real-time PCR

cDNA generation was carried out using the miScript Reverse Transcription Kit (QIAGEN). Real-time PCR detection of miRNAs and mRNAs was performed using the miScript SYBR-Green PCR Kit and QuantiTect and miScript Primer Assays (QIAGEN).

### RT–PCR

Reverse transcription was performed on 300 ng of total RNA using SuperScript VILO cDNA Synthesis Kit (Life Technologies) and PCR was performed using MyTaq HS DNA Polymerase (Bioline). Oligonucleotides are listed below.

>DMD E42F GAAGACATGCCTTTGGAAATTTCT

>DMD E43F CTACAACAAAGCTCAGGTCG

>DMD E46R CTCTTTTCCAGGTTCAAGTGG

>DMD E48R CTGAACGTCAAATGGTCCTTC

>DMD E53R TTGTACTTCATCCCACTGATT

>CELF2 E1aF CTATGAGAAATGAAGAGCTGC

>CELF2 E1bF GATTTCCTCCCGGACATGACG

>CELF2 E1cF CTCTGCTCGACAGCAGCACG

>CELF2 E2R GAGGACGTTGATCTGGTAGAC

>TMED2 F GACTCCAAAAATAGTGATGTTCAC

>TMED2 R GCTGTCATCGCCACTGCTAG

>CSDE1 F GCAATATTTGAGTATCACTGCG

>CSDE1 R AACCCCAGTTTCACGCAGTGC

### Western blot analysis

Protein extracts were loaded onto a NuPAGE Tris-Acetate Minigel 3–8% 1 mm (Invitrogen) or onto a NuPAGE Bis-Tris minigel 4–12% 1 mm (Invitrogen). Running and blotting were performed in an XCell SureLock Minicell (Invitrogen) according to the manufacturer's instructions and proteins were transferred to a nitrocellulose transfer membrane (Amersham Protran). Membranes were blocked with 10% non-fat dry milk and incubated o/n with primary antibodies. Protein detection was carried out with Clarity ECL Western Blotting Substrate (Bio-Rad). Primary antibodies: anti-dystrophin (NCL-DYS1 Novocastra Laboratories, 1:40); anti-myogenin (sc-12732 Santa Cruz 1:1,000); anti-MHC (anti-MF-20, 1:20), anti-MCK (sc-15161 Santa Cruz, 1:500 in TBST); anti-actinin (ACTN sc-15335 Santa Cruz, 1:1000); and anti-glyceraldehyde-3-phosphate dehydrogenase (GAPDH) (sc-365062 Santa Cruz, 1:10,000). Anti-CELF (ab50734 Abcam, 1:1,250). Anti-FLAG M2 (F1804 Sigma-Aldrich, 1:1,000). Secondary antibodies: ImmunoPure Goat Anti-Rabbit IgG Peroxidase Conjugated (31460 Pierce, 1:5,000); ImmunoPure Goat Anti-Mouse IgG Peroxidase Conjugated (31430 Pierce, 1:10,000); donkey anti-goat IgG-HRP (sc-2020 Santa Cruz, 1:5,000). Uncropped scans of blots in Fig. 1a,b,d are shown in Supplementary Fig. 4.

### Overexpression of CELF2a

Construct for the overexpression of CELF2a was obtained by cloning CELF2a coding sequence in pCDNA3.1(+) plasmid (Invitrogen). CELF2a coding sequence was amplified by PCR from WT myoblast cDNA using the oligonucleotides: 5′AAAA*AAGCTT*ATGCGCTGTCCCAAATCCGCT-3′ and 5′-AAAA*CTCGAG*TCAGTAAGGTTTGCTGTCGTTTTTG-3′.

The italicized bases are HindIII and XhoI restriction sites. pFLAG-CELF2a was obtained by inverse PCR using the oligos: 5′-gtccttgtagtccatAAGCTTAAGTTTAAACGCTAGC-3′ and 5′-gacgatgacaagATGCGCTGTCCCAAATCCGCTG-3′. The lower case bases are the ones coding for the FLAG tag.

### pLuc45 reporter construct and luciferase assays

The insert ‘45' was obtained by PCR from WT genomic DNA using the oligonucleotides: 5′-AAGGAAAAAA*GCGGCCGC*AAATACTTTGTTCATGTT-3′ and 5′-CC*TTAATTAA*ACAATCATTTATAATTGC -3′. The italicized bases are *Not*I and *Pac*I restriction sites. The insert, made of the whole exon 45 flanked by 391 bp of intron 44 and 347 bp of intron 45, was inserted into pcDNA3.1-Luc vector. Transfection efficiency of this construct was assessed by cotransfection of pRLTK plasmid (Promega) encoding for renilla luciferase gene. RLuc and FLuc activities were measured by Dual Glo Luciferase assay (Promega).

### RNA sequencing and bioinformatic analysis

Total RNA from GSΔ44, Δ44-1 and WT myoblasts and myotubes was isolated by Qiazol reagent and purified on miRNeasy columns according to manufacturer instructions (Qiagen). Ribosomal depletion and library preparation were carried out at Istituto di Genomica Applicata (Udine) according to the Illumina Truseq protocol. Reads (2 × 100 paired end) were produced on an Illumina HiSeq 2000. After base calling (Casava) and adaptor removal (Cutadapt), reads were alligned to the human genome (hg19) using TopHat 2.0.11 and the resulting bam files used as input for MATS 3.0.8. Ensembl GRCh37 was used as reference annotation. Exon skipping/inclusion events detected by MATS with both counting spliced reads and exon coverage were then filtered for false discovery rate<0.1 and concordance between the following pairwise comparisons: GSΔ44 versus WT (myoblasts, GM #A) with GSΔ44 versus Δ44-1 (myoblasts) and GSΔ44 versus WT (myotubes, DM#A) with GSΔ44 versus Δ44-1 (myotubes).

### CLIP assay

The RNP precipitation was performed using 5 × 10^6^ human WT and GSΔ44 myoblasts cultured in DM medium for 5 days, rinsed with PBS and UV crosslinked once at 400 mJ cm^−2^ in Spectrolinker XL-1000. Cell were harvested in 400 μl of PBS and 1.6 ml of Nuclear isolation buffer (0.32 M Sucrose; 10 mM Tris-HCl Ph 7.5; 5 mM MgCl_2_; and 1% Triton X-100) and incubated for 20 min on ice. Nuclei were collected by centrifugation at 2500*g* for 15 min and resuspended in 1 ml of RIP Buffer (150 mM KCl; 25 mM Tris-HCl Ph 7.4; 5 mM EDTA; 0.5 mM DTT; and 0.5% NP40). Nuclei were mechanically sheared using a dounce homogenizer and debris were eliminated by centrifugation. A total of 5 μg of Anti-CELF (ab50734 Abcam) and 5 μg of the control IgG antibodies were used for the immunoprecipitation for 12 h at 4 °C. A total of 40 μl of protein G agarose beads were added and incubated for 2 h at 4 °C. Beads were washed three times with RIP buffer and once with PBS. The beads were then treated with proteinase K (Ambion) before the addition of Tryzol for the RNA extraction. The oligonucleotides used in the RT–PCR analyses are listed below.

>DMD E10F GCAGTTCATTGATGGAGAGTG

>DMD E10 R CACCACTTCCACATCATTAGA

>DMD E44F GATTGGACAGATCTGTTGAGA

>DMD E45F ATGGCATTGGGCAGCGGCAAAC

>DMD E45R ACCTCCTGCCACCGCAGATTC

>IR F GCCATGTTGGCTAGGCTGGTCTC

>IR R GGCTTACACCACTGCGCTCAGC

>GAPDH F GGAAGGTGAAGGTCGGAGTC

>GAPDH R TTACCACAGTTAAAAGCAGCCC

### Statistical analyses

Statistical significance of differences between means was assessed by two-tailed *t*-test and *P*<0.05 was considered significant.

## Additional information

**Accession codes:** RNA sequencing reads were deposited at Gene Expression omnibus (GSE70389).

**How to cite this article:** Martone, J. *et al*. The lack of the Celf2a splicing factor converts a Duchenne genotype into a Becker phenotype. *Nat. Commun.* 7:10488 doi: 10.1038/ncomms10488 (2016).

## Supplementary Material

Supplementary Information Supplementary Figures 1-4, Supplementary Tables 1-2, Supplementary Methods and Supplementary References

Supplementary Data 1 Alternative splicing analysis of GS44 versus WT and 44-1 in GM and DM. The four spreadsheets include the raw output of MATS for the exon skipping events. All coordinates are hg19, annotation is Ensembl GRCh37.
