# Supplementary material for: The lack of the Celf2a splicing factor converts a Duchenne genotype into a Becker phenotype
Source: Nat Commun. 2016 Jan 22;7:10488. doi: 10.1038/ncomms10488 (PMC4736020; doi:10.1038/ncomms10488)
Supplement: Supplementary Information — Supplementary Figures 1-4, Supplementary Tables 1-2, Supplementary Methods and Supplementary References [file ncomms10488-s1.pdf]

Supplementary Figures

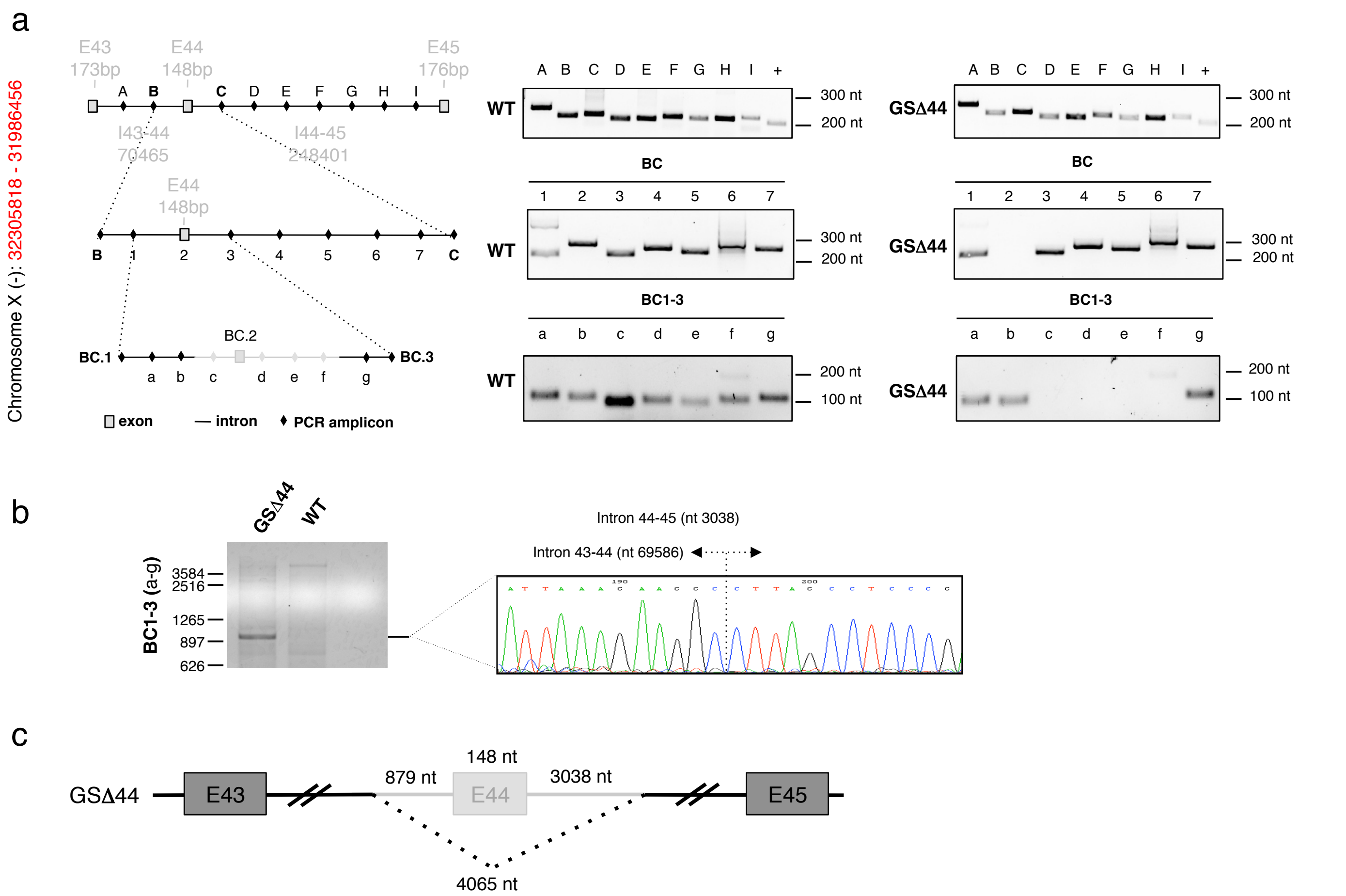

Supplementary Figure 1. Characterization of the GSΔ44 DMD mutation.

(**a**) Schematic representation of the PCR-based screening of GSΔ44 deletion: the region between exons 43 and 45 was divided into 10 regions of approximately the same length and two primers were designed at the boundaries of each region (amplicons A-I). Genomic DNA from WT and GSΔ44 cells was used to test the presence of each amplicon. Since GSΔ44 was previously reported as DMDΔ44 patient, the region between amplicons B and C was further sub-divided into 8 pieces and PCR primers were designed (amplicons 1-7). The resulting amplification pattern defined the deletion of GSΔ44 between amplicons 1 and 3. This region was divided into 8 pieces and primers were designed at the boundaries (amplicons a-g). The resulting amplification pattern defined the deletion of GSΔ44 between amplicons b and g. (**b**) The region identified as described above was amplified by using primers located externally. The exact length (4064 nt) and position of the deletion were therefore identified by Sanger sequencing and schematized in (**c**).

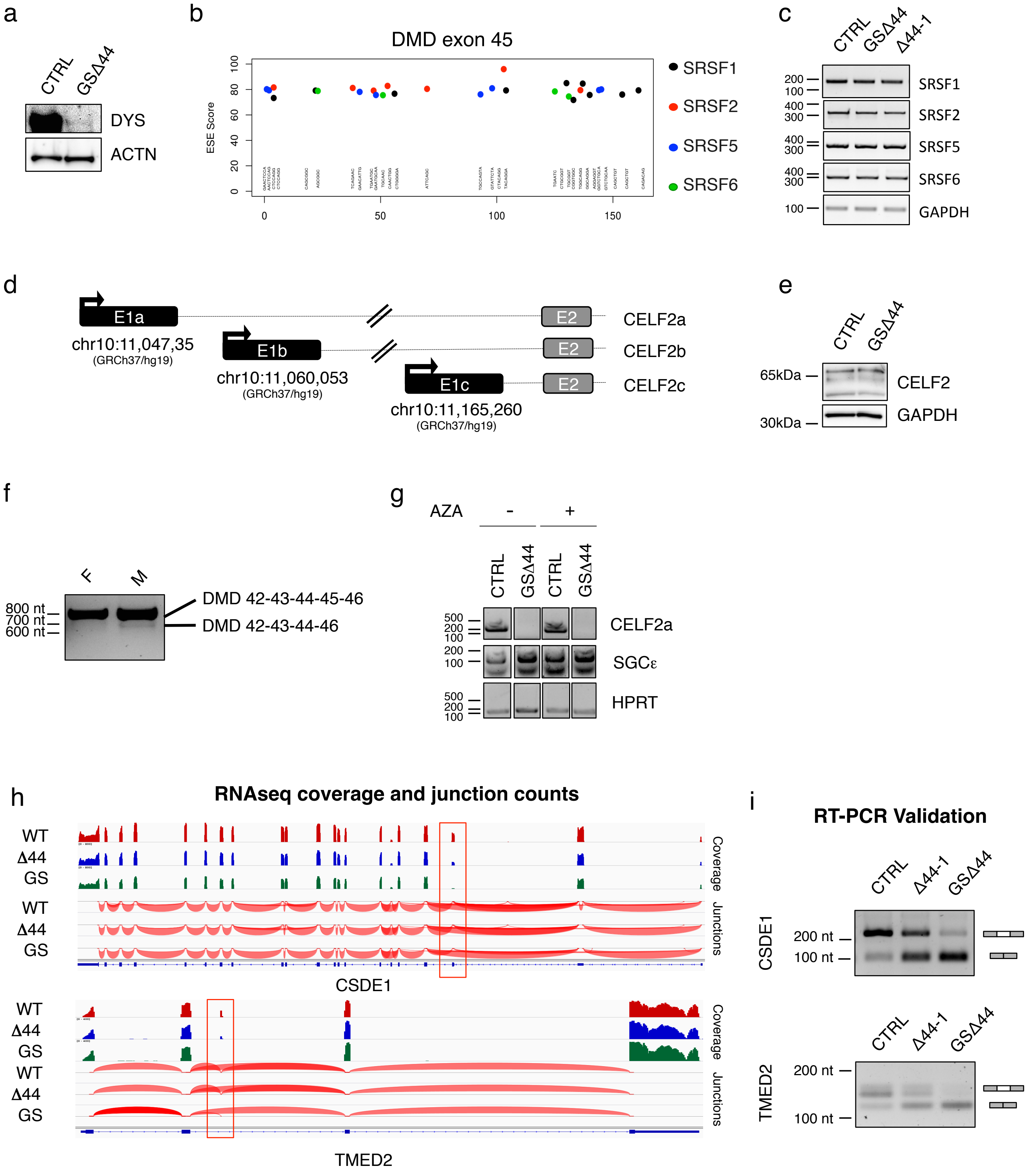

j

Patient	Mutation
BMD 3	Δ48-49
BMD 10	Point mutation
BMD 15	Δ48-53
BMD 16	Δ45-55

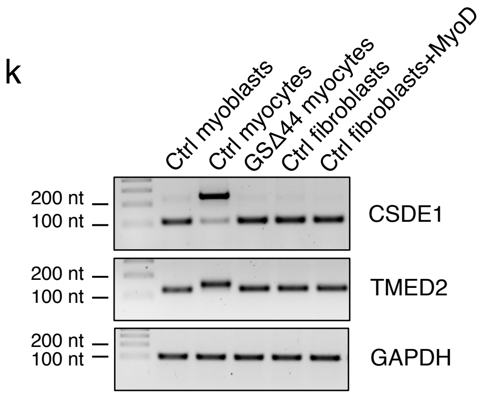

Supplementary Figure 2. Celf2 isoforms and alternative splicing in GSΔ44

(**a**) Western blot on proteins (20 µg) extracted from control (CTRL) and GSΔ44 fibroblasts trans-differentiated into myocytes via infection with a lentivirus containing a MyoD-expression cassette probed with antibodies against dystrophin (DYS). Actinin (ACTN) was used as a loading control. (**b**) Exonic splicing enhancer analysis of RNA motifs present in DMD exon 45. Predicted binding sites of SRSF1 (black), SRSF2 (red), SRSF5 (blue) and SRSF6 (green) are plotted together with their binding motif along the exon 45 sequence (x axys) with their normalized score obtained with Human Splicing Finder, selecting the ESE finder motifs[1,2]. (**c**) RT-PCR for the four splicing factors SRSF1, SRSF2, SRSF5 and SFSR6 on RNA from control (CTRL), GSΔ44 and Δ44-1 myocytes. GAPDH was used as control. (**d**) Schematic representation of the Celf2 locus: by using three different transcription start sites (TSS in exons 1a, 1b and 1c, black boxes), three main RNA and protein isoforms are produced, named Celf2a, Celf2b and Celf2c respectively. Coordinates corresponding to each TSS are shown. (**e**) Western blot on proteins (20 µg) extracted from MyoD-transduced control (CTRL) and GSΔ44 cells probed with antibodies against CELF2. GAPDH was used as a loading control. (**f**) RT-PCR performed on RNA from MyoD-transduced fibroblasts of GSΔ44 father (F) and GSΔ44 mother (M); 20 ng of cDNA were amplified with primers located in exons 42 and 46. (**g**) RT-PCR for CELF2A (top), SGCE (middle) and HPRT (bottom) mRNAs in WT and GSD44 cells untreated (-) and treated with 4 µM azacitidine for 7 days (+).

(**h**) Integrative Genomics Viewer (Broad Institute) coverage plot of the CSDE1 locus (upper panel) and TMED2 locus (lower panel) obtained from RNAseq analysis of WT (red), Δ44 (blue) and GSΔ44 (green) myotubes. Junction tracks for each sample are shown in red below the coverage plots: the thickness of the arcs is proportional to the coverage of spliced reads. Red blocks highlight the differentially spliced exons of CSDE1 and TMED2. (**i**) RT-PCR validation of the differentially spliced exons shown in (h) using RNA samples obtained from WT, Δ44-1 and GSΔ44 myocytes. The skipped/included exon is represented as a white box while the surrounding exons are represented as light grey boxes. (**j**) Table showing the mutations of BMD patients analyzed in Figure 2e. (**k**) RT-PCR for differentially spliced exons of CSDE1 and TMED2 on RNA from control myoblasts, control myocytes, GSΔ44 myocytes, control fibroblasts and MyoD-transduced control fibroblasts. GAPDH was used as control.

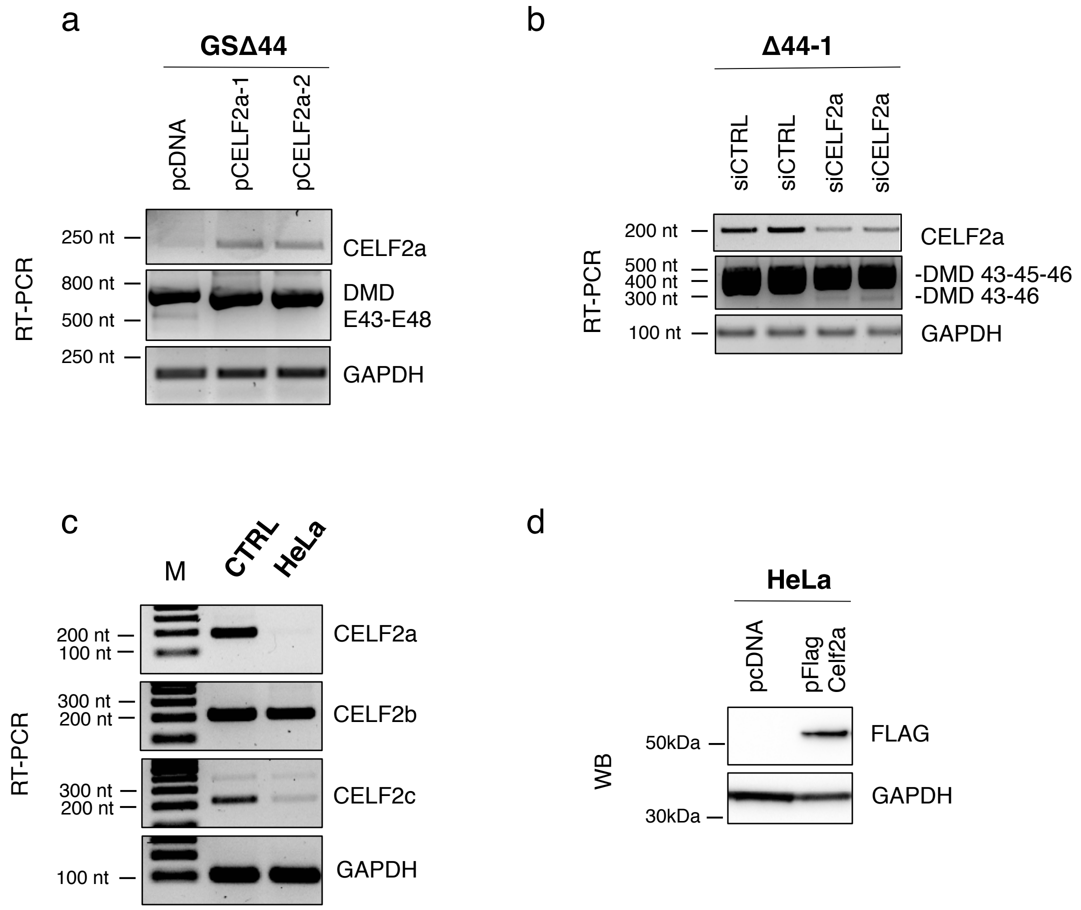

Supplementary Figure 3. CELF2a overexpression.

(**a**) MyoD-transduced GSΔ44 cells were transfected with an empty vector (pcDNA) or with CELF2a overexpressing vectors (pCELF2a-1 and pCELF2a-2). Cells were shifted to differentiation medium for 10 days and the RNA analyzed by RT-PCR for CELF2a expression and for DMD exon 45 skipping (DMD E44-E48). GAPDH was used as control. (**b**) RT-PCR on RNA from control Δ44-1 myocytes (siCTRL) and Δ44-1 myocytes after CELF2a depletion (siCELF2a). 50 ng of cDNA were amplified with primers for CELF2a expression and with primers located in exons 43 and 46 of the DMD gene for exon 45 skipping. GAPDH was used as control. (**c**) RT-PCR with oligos specific for the three Celf2 isoforms on RNA from control myoblasts (CTRL) and HeLa cells. GAPDH was used as control. M, molecular weight marker: GeneRuler™ 100bp (Thermo Scientific). (**d**) Western blot with anti-FLAG antibodies on proteins from HeLa cells transfected with an empty vector (pcDNA) or with a Flag-CELF2a construct (pFlag-CELF2a). GAPDH was used as loading control.

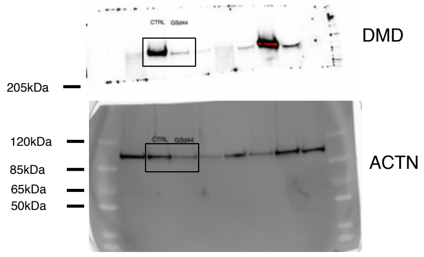

PageRuler™ Plus Prestained Protein Ladder
Gel: 3-8% SDS Tris-Acetate

Uncropped scans figure 1a

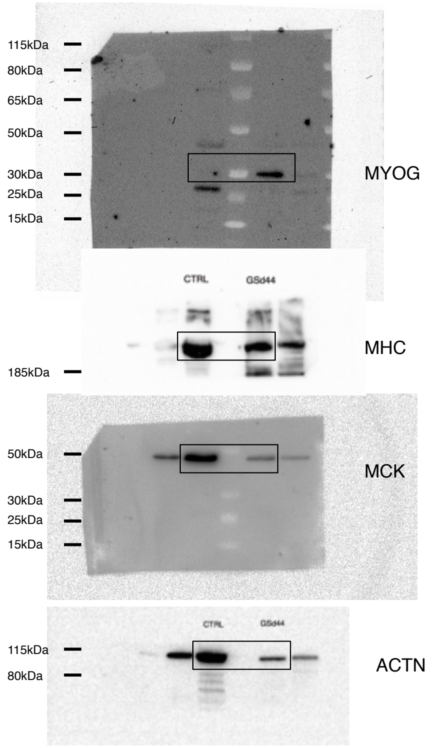

PageRuler™ Plus Prestained Protein Ladder
Gel: 4-12% SDS MOPS
Uncropped scans figure 1b

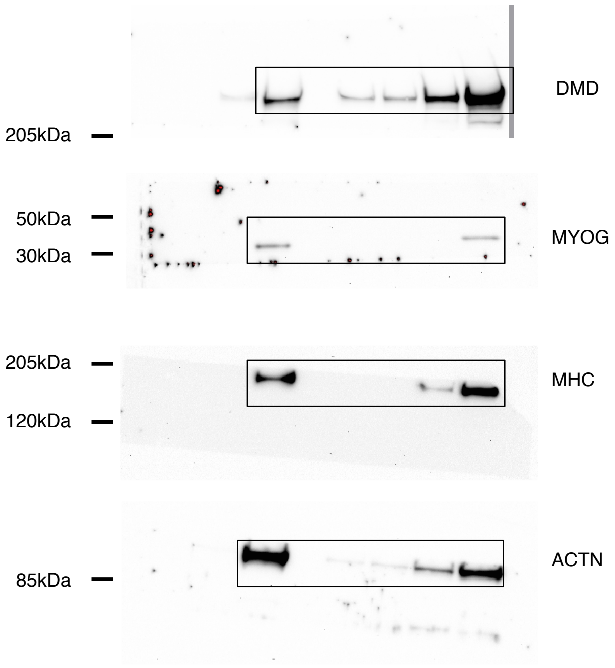

PageRuler™ Plus Prestained Protein Ladder
Gel: 3-8% SDS Tris-Acetate
Uncropped scans figure 1d

Supplementary Figure 4. Uncropped scans of the most important blots.

Uncropped scans of blots in figures 1a, b and d.

Age of the control	Gower's maneuver (in sec)	Four Stairs (in sec)	10 mt WALK (in sec)	NSAA	6MWT
3	2,22	6,56	5,87		
4	2,6	2,27	5,41		
5	1,63	4,69	5		
6	1,94	3,19	5,66		
7	1,73	2,13	4,96		
8	1,47	1,34	5,21		
9	3,65	1,99	4,4		
10	4,37	2,62	4,96		
11	5,34	4,06	7,73		
12	6,34	3,22	5,19	31/34	443,4
13	6,94	4,02	6,72	23/34	450
14	Not possible	1,00	10,1	21/34	373
15	Not possible	1,14	10,5	11/34	338
16	Not possible	2,00	9,34	13/34	302
17	Not possible	Not possible	9,54	8/34	270
18	Not possible	Not possible	8,19	8/34	252

Supplementary Table 1.

Clinical informations of GSΔ44 patient.

NSAA= North Star Ambulatory Assessment, 6MWT= 6-minute walk test[3].

	Sample A	Sample B	Type	N° events (Sample A: Sample B)
Myoblasts	GS Δ44	WT	SE	108 (63:45)
			MXE	33 (19:14)
			A5SS	3 (0:3)
			A3SS	14 (9:5)
			RI	55 (17:38)
	GS Δ44	Δ44-1	SE	33 (10:23)
			MXE	17 (9:8)
			A5SS	5 (0:5)
			A3SS	2 (0:2)
			RI	11 (5:6)
Myotubes	GS Δ44	WT	SE	83 (51:32)
			MXE	65 (36:29)
			A5SS	9 (1:8)
			A3SS	12 (5:7)
			RI	139 (8:131)
	GS Δ44	Δ44-1	SE	33 (10:23)
			MXE	17 (9:8)
			A5SS	5 (0:5)
			A3SS	2 (0:2)
			RI	11 (5:6)

LEGEND:
SE: Skipped exon
MXE: Mutually exclusive exon
A5SS: Alternative 5' splice site
A3SS: Alternative 3' splice site
RI: Retained intron

SKIPPED EXON in GSΔ44 versus both WT and Δ44-1

**Myoblasts**	
TFPI	tissue factor pathway inhibitor
ST7L	suppression of tumorigenicity 7 like
DCN	decorin
CD44	CD44 molecule (Indian blood group)
SLC4A7	solute carrier family 4, member 7
MAP4	microtubule-associated protein 4
PXN	paxillin
MYL6	myosin, light chain 6, smooth muscle and non-muscle
LGALS1	lectin, galactoside-binding, soluble, 1
ATP5SL	ATP5S-like
PRKAR1A	protein kinase, cAMP-dependent, type I, alpha
EIF4G2	eukaryotic translation initiation factor 4 gamma, 2
FN1	Fibronectin 1
CALD1	Caldesmon 1
RTN3	Reticulon 3
TANK	TRAF family member-associated NFKB activator
SULF1	Sulfatase 1
RPS24	ribosomal protein S24
PAM	peptidylglycine alpha-amidating monooxygenase
TMEM18	transmembrane protein 18
PLOD2	procollagen-lysine, 2-oxoglutarate 5-dioxygenase 2
COL6A3	collagen, type VI, alpha 3
USMG5	up-regulated during skeletal muscle growth 5 homolog
CD151	CD151 molecule (Raph blood group)
**Myotubes**	
CSDE1	cold shock domain containing E1, RNA-binding
TMED2	transmembrane emp24 domain trafficking protein 2
SULF1	Sulfatase 1
ITGB1	integrin, beta 1
TTN	titin

Supplementary Table 2. Alternative splicing analysis of WT, Δ44-1 and GSΔ44 cells.

Left panel shows the number of significant alternative splicing events in the indicated pairwise comparisons detected by MATS (by using both coverage of exons and spliced reads). The number of events is shown after the abbreviations explained in the legend. The right panel shows gene name and functional annotation of the genes marked by exon skipping events (SE) detected by MATS with FDR < 0.1 which differ concordantly between GSΔ44 and WT, GSΔ44 and Δ44-1, both in GM (top list) and DM (bottom list).

Supplementary Methods

PCR amplification of genomic DNA

PCR analysis of genomic DNA was carried out using Takara Ex Taq DNA Polymerase (ClonTech). 50ng of genomic DNA were used as template for each PCR reaction. Oligonucleotides are listed below.

>E44_F	CAGTGGCTAACAGAAGCTGAAC
>E44_R	GTTCAGCTTCTGTTAGCCACTG
>43-45.1_F	CCCTCTTGTGGGCCTGGAAC
>43-45.1_R	CAACAAATCCCCCTCTCACCCTG
>43-45.3_F	GCATAAGGGCTCTTGGATTAGGA
>43-45.3_R	CACTGAAGGGACTTGAAAGCACAC
>43-45.4_F	GTCTCCTTGGGATATCATTAAGAGC
>43-45.4_R	CCTCACGAGTGGGTTTGGTTCC
>43-45.5_F	CATAGGTTACTGAGGTGACGGAGG
>43-45.5_R	AGAAGGCTGATATTCAGGGAACAG
>43-45.6_F	GCTGGAGCAGGGAGCGGAGG
>43-45.6_R	GGTGGAGTTTCACCCTGTAGGC
>43-45.7_F	CGGAGGTAGGTATGTCACAGTTTG
>43-45.7_R	TGTGGACTCTGCTTACCAATCAAG
>43-45.A_F	GGGGAAATGAGCAGTTCTTGTGTC
>43-45.A_R	CCATCTTAACCATATCAGCTGCAC
>43-45.B_F	GAGTGTTCCATTTGGCTGCTGGA
>43-45.B_R	GCCATGCCTATCCCTGAGTACAT
>43-45.C_F	CAGTTGGGTGATTAGGAGAGGTG
>43-45.C_R	GTTAGCTTCAGGTCTGCCTTCC
>43-45.D_F	GCAGTTAGAGAGTCGGGAAGAAG
>43-45.D_R	GATAGAGCCTGGTACTTGAGGTG
>43-45.E_F	GGATACCGATGGTTGAAAGTGCC
>43-45.E_R	GAGACAGATAGTGGTGCCAGGG
>43-45.F_F	GGAAGATTTCTCTAAAGACCAAAGGC
>43-45.F_R	GCAGAAACAGCTATGCAGTGGAG
>43-45.G_F	CCGTTGCAACTGTTGTCTGAATG
>43-45.G_R	TTGTGTATCAACGGCAGTGTGAG
>43.45.H_F	GAGGACTTGAGGCTACCAACCAG
>43-45.H_R	CCACTTTATGAGGGATCATGTGTCC
>43-45.I_F	CTTAGGAAGGGTGTAACATTCTCTGT
>43-45.I_R	TGAATATGAGGATACATCGACAGGG
>43-45.a_F	GGCAAGATCATTTTGTTGTGG
>43-45.a_R	GAGGTATTCTCTAACTGTAG
>43-45.b_F	CCAGTTGATTCTTATGTGCAAC
>43-45.b_R	CAAGCATTTGGTCACCTTCC
>43-45.c_F	GCCAATAGTCCAAAATAGTTGC
>43-45.c_R	ATGGAAGGTTGCAATTTTTCCC
>43-45.d_F	GAAGCATCGTAACAGCAAGGTG
>43-45.d_R	ATGCCACAAGTTCTCCTTCTG
>43-45.e_F	GTACCTCCATTCTACTCTTTG
>43-45.e_R	TACAATGGCTTTCCAAGAAACC
>43-45.f_F	TCTCTGGTATTTTGCCCTGTG

>43-45.f_R	ATTGAAGGACAGTGATCCTTG
>43-45.g_F	GATGTAGACAGTGGCTGTTAG
>43-45.g_R	GTGCTTCTGCGTGTGTTTG

CELF2a depletion in Δ44-1 myocytes

Δ44-1 myoblasts were infected with lentiviral particles containing a short hairpin RNA (shRNA) expression cassette against CELF2a (pLKO-shCELF2a) or a shRNA scramble as control (pLKO-shC002 from SIGMA) to generate a long-term knockdown of CELF2a gene expression.

To obtain pLKO-shCELF2a expressing lentiviral vector the following oligos, designed to contain restriction enzyme-like cleavage ends after annealing, were used:

siCELF2a NdeI for: TATG CTT ACC GTA ACT TGA AAG TAT TTC GAT TTC TTG GCT TTA TAT ATC TTG TGG AAA GGA CGA AAC ACC GGG CAT TGA TGT TTG AGC ATA TTT CAA GAG A ATA TGC TCA AAC ATC AAT GCT TTT TG-3'

siCELF2a EcoRI rev: AATTC AAA AAG CAT TGA TGT TTG AGC ATA TTC TCT TGA AAT ATG CTC AAA CAT CAA TGC CCG GTG TTT CGT CCT TTC CAC AAG ATA TAT AAA GCC AAG AAA TCG AAA TAC TTT CAA GTT ACG GTA AG CA-3'

The pLKO plasmid encodes for puromycin resistance, thereby puromycin 0.7μg ml^{-1} was added to select cells stably expressing shRNA against CELF2a. After four days cells were transfected with LNA™ GapmeRs antisense oligonucleotides against CELF2a (Exiqon GGATTTGGGACAGCGC) or a scramble sequence. Transfection of LNA™ GapmeRs was carried out with the use of Lipofectamine-2000 (Invitrogen) according to the manufacturer's specifications. The day after cells were switched to differentiation medium. Cells were harvested 10 days after differentiation induction.

Azacytidin treatment

WT and GSΔ44 cells were cultured for 7 days in growth medium containing 4 μM azacitidine. SGCE was a positive control[4]. Oligonucleotides are listed below.

>SGCe_F	GTTTTGGGTAAGGTGGAAATTC
>SGCe_R	ACCACTGGCACATTCTTGCTG

Oligo used in RT-PCR of Supplementary Figure 2c

>SRSF1 F	CGCGACGGCTATGATTACGA
>SRSF1 R	CCAACTTCCACTTGGAGGCA
>SRSF2 F	CTT CGT TCG CTT TCA CGA CAA
>SRSF2 R	AGA CGA GGA CTT GGA CTT GG
>SRSF5 F	TGGCTGTCGGGTATTCATCG

>SRSF5 R	ACAGGTGGAGCATTTCGTCT
>SRSF6 F	TTACGAGCTGAACGGCAAGG
>SRSF6 R	GTGGGCATCCGCATAGGTTA

Supplementary References

1. Cartegni, L., Wang, J., Zhu, Z., Zhang, M. Q. & Krainer, A. R. ESEfinder: A web resource to identify exonic splicing enhancers. *Nucleic Acids Res.* **31,** 3568–3571 (2003).
2. Desmet, F.-O. *et al*. Human Splicing Finder: an online bioinformatics tool to predict splicing signals. *Nucleic Acids Res.* **37,** e67–e67 (2009).
3. Mazzone, E. *et al*. North Star Ambulatory Assessment, 6-minute walk test and timed items in ambulant boys with Duchenne muscular dystrophy. *Neuromuscul. Disord.* **20,** 712–716 (2010).
4. Grabowski, M. *et al.* The epsilon-sarcoglycan gene (SGCE), mutated in myoclonus-dystonia syndrome, is maternally imprinted. *Eur J Hum Genet.* **11(2),** 138-44 (2003).
